# Localization of Puncta Adherentia Junctions at GABAergic Synapses on Parvalbumin‐Positive GABAergic Inhibitory Neurons in the Mouse Hippocampus

**DOI:** 10.1002/cne.70152

**Published:** 2026-03-19

**Authors:** Kimitaka Katanazaka, Hajime Shiotani, Muneaki Miyata, Takeshi Kameyama, Shin Kedashiro, Ryouhei Komaki, Shota Nishii, Yutaro Kashiwagi, Yuka Sato, Shigeo Okabe, Norio Chihara, Riki Matsumoto, Kiyohito Mizutani, Yoshimi Takai

**Affiliations:** ^1^ Division of Pathogenetic Signaling, Department of Psychiatry Kobe University Graduate School of Medicine Kobe Hyogo Japan; ^2^ Division of Neurology Kobe University Graduate School of Medicine Kobe Hyogo Japan; ^3^ Division of Pathogenetic Signaling, Institute of Advanced Medical Sciences Tokushima University Tokushima Japan; ^4^ Department of Cellular Neurology, Graduate School of Medicine The University of Tokyo Tokyo Japan; ^5^ RIKEN Center for Brain Science Wako Saitama Japan; ^6^ Department of Neurology Kyoto University Graduate School of Medicine Kyoto Japan

**Keywords:** afadin, catenin, GABAergic synapses, glutamatergic synapses, nectin, N‐cadherin, puncta adherentia junctions (PAJs)

## Abstract

Puncta adherentia junctions (PAJs) have been observed at glutamatergic excitatory synapses on glutamatergic excitatory neurons (*E*→*E* synapses) in the mouse hippocampus and are considered to be the mechanical adhesion sites between axon terminals and dendritic shafts. However, it remains unclear whether they are present at other types of synapses, such as glutamatergic synapses on GABAergic inhibitory neurons (*E*→*I* synapses) and GABAergic synapses on excitatory neurons (*I*→*E* synapses) and inhibitory neurons (*I*→*I* synapses). We showed here that the PAJ components, such as nectin‐1, nectin‐3, l‐afadin, N‐cadherin, β‐catenin, and αN‐catenin, were observed at all four types of synapses in cultured mouse hippocampal neurons. In the adult mouse hippocampus, these PAJ components were observed at a subset of *I*→*I* synapses on parvalbumin‐positive GABAergic inhibitory neurons, similarly to their presence at *E*→*E* synapses in the CA1 and CA3 regions. In contrast, several PAJ components, including nectin‐3, l‐afadin, N‐cadherin, β‐catenin, and αN‐catenin, were observed at *E*→*I* and *I*→*E* synapses, whereas nectin‐1 was absent. These results indicate that the PAJs are formed at a subset of *I*→*I* synapses in addition to *E*→*E* synapses, where all examined PAJ components are present, whereas *E*→*I* and *I*→*E* synapses exhibit only partial localization of these components, reflecting molecular diversity among different synapses in the adult mouse hippocampus.

## Graphical abstract

Puncta adherentia junctions (PAJs) were observed not only at glutamatergic excitatory synapses on excitatory neurons (*E*→*E* synapses) but also at glutamatergic synapses on GABAergic inhibitory neurons (*E*→*I* synapses) and at GABAergic synapses on excitatory (*I*→*E* synapses) and inhibitory neurons (*I*→*I* synapses) in cultured mouse hippocampal neurons. In the adult hippocampus, PAJs were present at all *E*→*E* synapses and a subset of *I*→*I* synapses on parvalbumin‐positive GABAergic neurons, showing molecular diversity among hippocampal synapse types.

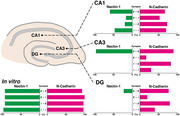

## 1 Introduction

Puncta adherentia junctions (PAJs) are specialized adhesive structures found at synapses, serving as mechanical adhesion sites between axon terminals and dendritic shafts. Unlike synaptic junctions (SJs), which are associated with synaptic vesicles and function as neurotransmission sites, the PAJs are not involved in synaptic vesicle release. Instead, they play a role in maintaining the structural integrity of synapses by facilitating mechanical attachment between pre‐ and postsynaptic elements (Peters et al. 1991).

The PAJs were first observed electron microscopically as a symmetrical, paramembranous dense structure formed between the mossy fiber terminal buttons and the pyramidal cell dendritic shafts in the CA3 region (CA3) of the rabbit hippocampus (Hamlyn 1962). Subsequent studies identified the PAJs at excitatory synapses in the dentate gyrus (DG) of the rat hippocampus (Amaral and Dent 1981; Anthes and Petit 1995) and at Schaffer collateral excitatory synapses in the CA1 region (CA1) of the mouse hippocampus (Špaček and Harris 1997, 1998). Over time, the presence of the PAJs has also been noted in several other brain regions, such as the rat lateral vestibular nucleus (Sotelo and Palay 1970), the rat thalamus (Lieberman and Špaček 1997; Špaček and Lieberman 1974), and the mouse visual cortex. Additionally, the PAJs have been observed in the human frontal, temporal, and parietal cortices (Špaček 1985, 1987).

The PAJs share molecular and functional similarities with epithelial adherens junctions (AJs), which are circumferentially continuous and belt‐like, forming a continuous band around epithelial cells to maintain cell‐cell adhesion and tissue integrity. Epithelial AJs play critical roles in regulating epithelial sheet morphology, establishing mechanical connections between neighboring cells and facilitating the formation of tight junctions (TJs) at the apical side of the AJs to form the apical junctional complex (Gumbiner et al. 1988; Harris and Tepass 2010). The components of epithelial AJs, such as cell adhesion molecule (CAM) E‐cadherin, along with β‐catenin and αE‐catenin, have been extensively studied for their roles in regulating cell‐cell adhesion and maintaining tissue integrity (Takeichi 1977, 2014). E‐Cadherin binds to β‐catenin, which in turn associates with αE‐catenin, a protein that binds to the actin cytoskeleton, reinforcing the mechanical strength of the adhesion. Nectin, another CAM, is a family consisting of four members: nectin‐1, nectin‐2, nectin‐3, and nectin‐4 (Takai et al. 2008). These molecules interact to promote cell adhesion, with nectins binding afadin, which in turn connects to the actin cytoskeleton (Takai et al. 2008). Our recent study revealed that environmental cues, such as lysophosphatidic acid, can influence the organization of the apical junctional complex, highlighting the dynamic regulation of the AJs and TJs in epithelial cells (Sakakibara et al. 2022).

In contrast to epithelial AJs, neuronal PAJs are punctate and localized at specific sites between axon terminals and dendritic shafts, rather than forming continuous, belt‐like structures. Thus, PAJs differ from epithelial AJs not only in their localization and morphology but also in their specific functions. Nevertheless, several molecular components are shared between the two junction types. For example, N‐cadherin, β‐catenin, and αN‐catenin were first identified as the PAJ components in the mouse cerebellar cortex (Uchida et al. 1996). We subsequently identified that nectin‐1, nectin‐3, and afadin are also to be the PAJ components at mouse hippocampal mossy fiber synapses (Mizoguchi et al. 2002; Nishioka et al. 2000). Although the precise mechanisms by which PAJs are formed remain unclear, current evidence suggests that their assembly may involve molecular processes similar to those governing epithelial AJ formation (Mizutani et al. 2021).

In the nervous system, glutamatergic excitatory neurons and γ‐aminobutyric acidergic (GABAergic) inhibitory interneurons form neural networks through four types of synapses. In this study, these are referred to simply as excitatory neurons and inhibitory neurons, respectively. Accordingly, four types of synapses can be distinguished: glutamatergic excitatory synapses on excitatory neurons (*E*→*E* synapses), glutamatergic excitatory synapses on inhibitory neurons (*E*→*I* synapses), GABAergic inhibitory synapses on excitatory neurons (*I*→*E* synapses), and GABAergic inhibitory synapses on inhibitory neurons (*I*→*I* synapses) (Komaki et al. 2025). These synapses are crucial for maintaining excitation and inhibition (E/I) balance within neural circuits, which is essential for normal brain function and the regulation of processes such as learning, memory, and behavior (Zhou and Yu 2018).

In the hippocampus, excitatory neurons form a trisynaptic circuit through *E*→*E* synapses, with granule cells in the DG region, and the pyramidal cells in the CA3 and the CA1, being the predominant excitatory neurons (Andersen et al. 1971; Johnston and Amaral 2004). Inhibitory neurons also play a critical role in shaping these circuits, with various types of inhibitory neurons found in the CA1 and the CA3, including parvalbumin (PV)‐positive basket cells, axo‐axonic cells (also called chandelier‐type cells), and bistratified interneurons (Freund and Buzsáki 1996; Klausberger and Somogyi 2008; Sik et al. 1995). These interneurons regulate both excitation and inhibition, thereby contributing to the balance of neural activity essential for normal hippocampal function.

Mossy fiber and Schaffer collateral synapses are representative *E*→*E* synapses in the hippocampal trisynaptic circuit. However, it remains unclear whether the PAJs are also formed at other types of synapses, such as *E*→*I*, *I*→*E*, and *I*→*I* synapses. In this study, we examined the localization of PAJ components not only at *E*→*E* synapses but also at the other three types of synapses, using cultured mouse hippocampal neurons and the mouse hippocampus. In cultured neurons, PAJ components were observed at all four types of synapses. In contrast, in the adult mouse hippocampus, PAJ components were observed at a subset of *I*→*I* synapses, similarly to their presence at *E*→*E* synapses in the CA1 and CA3 regions, whereas *E*→*I* and *I*→*E* synapses exhibited only partial localization of these components, highlighting the diversity of adhesive structures across different synapses.

## 2 Materials and Methods

### 2.1 Animals

C57BL/6J mice were purchased from CLEA Japan. Postnatal day (P) 0 was defined as the day of birth. All animal experiments were performed in accordance with institutional guidelines and approved by the administrative panel on laboratory animal care of Kobe University. This study was approved by the president of Kobe University after being reviewed by the Kobe University Animal Care and Use Committee (Approval No. 30‐06‐01, 30‐07‐01, and P230503), and animal experiments were conducted in accordance with regulations for animal experimentation of Kobe University.

### 2.2 Cell Culture

Mouse hippocampal neurons for culture were prepared as described previously (Toyoshima et al. 2014). In brief, hippocampal neurons dissociated with trypsin were plated at a density of 3 × 10^4^ cells/cm^2^ on poly‐L‐lysine‐coated coverslips in Minimum Essential Medium (Thermo Fisher Scientific) with 10% fetal bovine serum and cultured at 37°C in a humidified 5% CO_2_ incubator. After a 3 h incubation, all medium was replaced with Neurobasal Medium (Thermo Fisher Scientific) containing B‐27 Supplement (Thermo Fisher Scientific) and GlutaMAX (Thermo Fisher Scientific). Cytosine arabinoside (1 µM) was added to the cultures after 2 days to inhibit glial proliferation.

### 2.3 Antibody (Ab) Characterization

The primary Abs used in this study are listed in Table 1. They were characterized as follows. The Abs listed below were purchased from commercial sources. Guinea pig anti‐l‐afadin polyclonal Ab (pAb) (Frontier Institute, Cat# Afadin‐GP‐Af690, RRID: AB_2858199) was raised against a 33‐mer synthetic peptide KERQRLFSQGQDVSDKVKASRKLTELENELNTK corresponding to mouse l‐afadin (amino acids 1,788–1,820). Rabbit anti‐l‐afadin pAb (Frontier Institute, Cat# Afadin‐Rb‐Af900, RRID: AB_2858198) was raised against a 33‐mer synthetic peptide KERQRLFSQGQDVSDKVKASRKLTELENELNTK corresponding to mouse l‐afadin (amino acids 1,788–1,820). Rabbit anti‐bassoon monoclonal Ab (mAb) (Cell Signaling, Cat# 6897, RRID: AB_10828496) recognized a 420‐kDa band in western blotting of mouse and rat brain lysate (manufacturer's information). Rabbit anti‐N‐cadherin mAb (Cell Signaling, Cat# 13116, RRID: AB_2687616) recognized a 140‐kDa band in western blotting of mouse and rat brain lysate (Zhao et al. 2020). Rabbit anti‐αN‐catenin pAb (Synaptic Systems, Cat# 281 103, RRID: AB_2620014) was raised against a 19‐mer synthetic peptide PEEFQTRVRRGSQKKHISP corresponding to mouse αN‐catenin (amino acids 921–939). The pAb mainly recognized a 100‐kDa band in western blotting of rat brain lysate (manufacturer's information). Rat anti‐αN‐catenin mAb (Developmental Studies Hybridoma Bank, NCAT2, RRID: AB_528123), developed by Dr. Masatoshi Takeichi, recognized 113‐kDa and 102‐kDa bands in western blotting of chicken whole brain lysate (Hirano et al. 1992). Specificity was verified by western blotting of the immunoprecipitates obtained using NCD‐2 mAb from chicken brain (Hirano et al. 1992). Mouse anti‐β‐catenin mAb (Thermo Fisher Scientific, Cat# 13–8400, RRID: AB_2533039) recognized an 86‐kDa band in western blotting of mouse brain (Lei et al. 2022). Specificity was verified by immunoreactivity in conditional ablation of *β‐catenin* gene in mouse hippocampal neurons (Bamji et al. 2003). Rabbit anti‐β‐catenin mAb (Cell Signaling, Cat# 8480, RRID: AB_11127855) was raised against a synthetic peptide corresponding to residues surrounding Pro714 of human β‐catenin protein. The mAb recognized an 85‐kDa band in western blotting of mouse brain (Del Grosso et al. 2022). Specificity was verified by western blotting of the *β‐catenin*‐knockdown cultured JHH5 cells (Ding et al. 2017). Biotin‐conjugated mouse anti‐GAD67 mAb (EMD Millipore, Cat# MAB5406B, RRID: AB_2938602) recognized a 67‐kDa band in western blotting of mouse brain (Pappas et al. 2015). Specificity was verified by western blotting of the *GAD67*‐knockdown primary cultured mouse hippocampal neurons (Heldt et al. 2012). Mouse anti‐gephyrin mAb (Synaptic Systems, Cat# 147 011, RRID: AB_887717) was raised against a native protein corresponding to rat gephyrin (amino acids 1–768). The mAb recognized a 95‐kDa band in western blotting of mouse brain lysate (Paul et al. 2012). Specificity was verified by immunofluorescence staining of the *gephyrin*‐deficient mouse brain (Feng et al. 1998). Mouse anti‐homer1 mAb (Synaptic Systems, Cat# 160 011, RRID: AB_2120992) was raised against a synthetic peptide corresponding to the N‐terminal half of human Homer 1 (manufacturer's information). The mAb recognized a 56‐kDa band in western blotting of mouse brain (Yoo et al. 2020). Specificity was verified by sandwich enzyme‐linked immunosorbent assay of brain and liver (Soria Van Hoeve and Borst 2010). Chicken anti‐MAP2 pAb (Abcam, Cat# ab5392, RRID: AB_2138153) recognized two bands at approximately 280‐kDa (MAP2a and MAP2b isoforms) and 70‐kDa (MAP2c) in western blotting of rat brain (manufacturer's information). Specificity was verified by immunofluorescence staining of the mouse cultured glia (An et al. 2012). Rat anti‐nectin‐1 mAb (MBL International, Cat# D146‐3, RRID: AB_590847) recognized a 90‐kDa band in western blotting of mouse brain (Miyata et al. 2016; Shiotani et al. 2017). Specificity was verified by western blotting of the *nectin‐1*‐deficient mouse brain (Honda et al. 2006). Rat anti‐nectin‐3 mAb (MBL International, Cat# D084‐3, RRID: AB_592587) recognized a 90‐kDa band in western blotting of mouse brain (Miyata et al. 2016; Satoh‐Horikawa et al. 2000; Shiotani et al. 2017). Specificity was verified by western blotting of the *nectin‐3*‐deficient mouse brain (Honda et al. 2006). Goat anti‐PV pAb (Frontier Institute, Cat# PV‐Go‐Af460, RRID: AB_2571614) recognized a 13‐kDa band in western blotting of mouse brain (manufacturer's information). This pAb was verified to selectively label the subpopulation of GAD‐positive inhibitory neurons (Nakamura et al. 2004). Guinea pig anti‐VGAT pAb (Frontier Institute, Cat# VGAT‐GP‐Af1000, RRID: AB_2571624) was raised against mouse VGAT (amino acids 81–112). Rabbit anti‐VGAT pAb (Frontier Institute, Cat# VGAT‐Rb‐Af500, RRID: AB_2571622) was raised against mouse VGAT (amino acids 81–112). The pAb recognized a 57‐kDa band in western blotting of mouse brain (Fukudome et al. 2004). Specificity was verified by immunofluorescence staining of GABAergic neurons (Miura et al. 2006). Guinea pig anti‐VGLUT1 pAb (EMD Millipore, Cat# AB5905, RRID: AB_2301751) recognized a 65‐kDa band in western blotting of rat brain (Chen et al. 2016). Specificity was verified by the absence of immunostaining using the Ab that was pre‐absorbed with the immunogen peptide (EMD Millipore, Cat# AG208) (manufacturer's information). Rabbit anti‐VGLUT1 pAb (Frontier Institute, Cat# VGluT1‐Rb‐Af500, RRID: AB_2571616) was raised against mouse VGluT1 (amino acids 531–560). The pAb recognized a 60‐kDa band in western blotting of mouse brain (manufacturer's information). Primary Abs were visualized using goat or donkey fluorochrome‐conjugated secondary Abs and Streptavidin‐conjugated Alexa Fluor 750 (Thermo Fisher Scientific). The fluorochromes used were Alexa Fluor 405, 488, 555, 568, 647, and 750 (Thermo Fisher Scientific).

**TABLE 1:** Primary antibodies (Abs).

Ab name, species	Immunogen	Manufacturer, catalog number, RRID	Dilution
Guinea pig anti‐l‐afadin pAb	Synthetic peptide: KERQRLFSQGQDVSDKVKASRKLTELENELNTK, corresponding to amino acids 1788–1820 of mouse l‐afadin	Frontier Institute, Afadin‐GP‐Af690 RRID: AB_2858199	1:300
Rabbit anti‐l‐afadin pAb	Synthetic peptide: KERQRLFSQGQDVSDKVKASRKLTELENELNTK, corresponding to amino acids 1788–1820 of mouse l‐afadin	Frontier Institute, Afadin‐Rb‐Af900 RRID: AB_2858198	1:300
Rabbit anti‐bassoon mAb	Synthetic peptide: corresponding to residues surrounding Gln1217 of human bassoon protein	Cell Signaling Technology, 6897 RRID: AB_10828496	1:300
Rabbit anti‐N‐cadherin mAb	Synthetic peptide: corresponding to residues surrounding Arg526 of human N‐cadherin protein	Cell Signaling Technology, 13116 RRID: AB_2687616	1:300
Rabbit anti‐αN‐catenin pAb	Synthetic peptide: PEEFQTRVRRGSQKKHISP, corresponding to amino acids 921–939 of mouse αN‐catenin	Synaptic Systems, 281 103 RRID: AB_2620014	1:300
Rat anti‐αN‐catenin mAb	An isolated 113‐kDa and 102‐kDa protein obtained by immunoprecipitation with N‐cadherin from detergent extracts of the cultured 10‐day‐old chick embryonic brain cells	Hirano et al. (1992) Developmental Studies Hybridoma Bank, NCAT2 RRID: AB_528123	1:300
Mouse anti‐β‐catenin mAb	Synthetic peptide: consisting of the maltose binding protein fused to a 100 amino acid segment of the C‐terminus of chicken beta‐Catenin	Thermo Fisher Scientific, 13‐8400 AB_2533039	1:300
Rabbit anti‐β‐catenin mAb	Synthetic peptide: corresponding to residues surrounding Pro714 of human β‐catenin protein	Cell Signaling Technology, 8480 RRID: AB_11127855	1:300
Biotin‐conjugated mouse anti‐GAD67 mAb	Recombinant GAD67 protein	EMD Millipore, MAB5406B RRID: AB_2938602	1:100
Mouse anti‐gephyrin mAb	Native protein corresponding to rat gephyrin, amino acids 1‐768	Synaptic Systems, 147 011 RRID: AB_887717	1:300
Mouse anti‐homer1 mAb	Recombinant protein corresponding to the N‐terminal half of human homer1	Synaptic Systems, 160 011 RRID: AB_2120992	1:100
Chicken anti‐MAP2 pAb	Recombinant fragment corresponding to human MAP2. Mix of recombinant human constructs of projection domain sequences, amino acids 235–1588	Abcam, ab5392 RRID: AB_2138153	1:1000
Rat anti‐nectin‐1 mAb	The fusion protein of the extracellular region of mouse nectin‐1 (amino acids 28–345) with IgG Fc	MBL International, D146‐3 RRID: AB_590847	1:300
Rat anti‐nectin‐3 mAb	The fusion protein of the extracellular region of mouse nectin‐3 (amino acids 54–400) with IgG Fc	Satoh‐Horikawa et al. (2000) MBL International, D084‐3 RRID: AB_592587	1:300
Goat anti‐PV pAb	Mouse PV, whole sequence	Frontier Institute, PV‐Go‐Af460 RRID: AB_2571614	1:300
Guinea pig anti‐VGAT pAb	Mouse VGAT, amino acids 31–112 (BC052020)	Frontier Institute, VGAT‐GP‐Af1000 RRID: AB_2571624	1:1000
Rabbit anti‐VGAT pAb	Mouse VGAT, amino acids 31–112 (BC052020)	Frontier Institute, VGAT‐Rb‐Af500 RRID: AB_2571622	1:1000
Guinea pig anti‐VGLUT1 pAb	Synthetic peptide from rat VGLUT1 protein with no overlap to VGLUT2	EMD Millipore, AB5905 RRID: AB_2301751	1:1000
Rabbit anti‐VGLUT1 pAb	Mouse VGluT1, C‐terminal 531–560 amino acids (BC054462)	Frontier Institute, VGluT1‐Rb‐Af500 RRID: AB_2571616	1:1000

### 2.4 Immunofluorescence Microscopy

Immunofluorescence microscopy was performed as described previously (Nozawa et al. 2023). In brief, cultured neurons on coverslips were fixed in 2% paraformaldehyde (PFA) in phosphate‐buffered saline (PBS) or 2% formaldehyde in Neurobasal Medium at room temperature (RT) for 15 min and 0.25% Triton X‐100 in PBS at RT for 10 min. They were then incubated with a blocking buffer (10% normal goat or donkey serum, 1% bovine serum albumin [BSA], 0.25% Triton X‐100 in PBS) at RT for 30 min. Samples were stained with the indicated Abs overnight at 4°C and then with appropriate fluorophore‐conjugated secondary Abs.

Immunofluorescence microscopy of frozen brain sections was performed as described previously (Nozawa et al. 2023). In brief, mice were deeply anesthetized and transcardially perfused at RT with 1× Hanks’ balanced salt solution (HBSS) with Ca^2+^ and Mg^2+^ (Thermo Fisher Scientific) containing 10 mM HEPES, 1 mM sodium pyruvate, 4% sucrose, heparin, and a protease inhibitor cocktail (cOmplete Mini; Roche Diagnostics), followed by perfusion with 2% PFA in the aforementioned HBSS‐based buffer. After dehydration with 30% sucrose in HBSS, whole brains were embedded in OCT Compound (Sakura Finetek). Cryostat sections were incubated at 62°C for 20 min in HistoVT One antigen retrieval solution (Nacalai Tesque) and then incubated with 1% BSA, 10% normal goat or donkey serum, and 0.25% Triton X‐100 in PBS at RT for 30 min. Sections were stained with the indicated Abs, and then with appropriate fluorophore‐conjugated secondary Abs.

Fluorescence microscopic image analyses were performed on a BZ‐X710 All‐in‐One Fluorescence Microscope (KEYENCE) using a Plan Apochromat 20×/0.95 numerical aperture objective lens (Nikon). Images captured on the BZ‐X710 were analyzed using BZ‐X‐Analyzer software (KEYENCE). Structured illumination microscopic (SIM) image analyses were performed on an ELYRA PS.1 microscope (Carl Zeiss) using a Plan Apochromat 100×/1.46 numerical aperture oil immersion objective lens (Carl Zeiss). Images were then reconstructed using ZEN software (Carl Zeiss) based on the structured illumination algorithm.

### 2.5 Quantification of PAJ Component Localization at Different Synapse Types

To estimate the proportion of synapses exhibiting PAJ component localization, approximately 30 synapses per synapse type were randomly selected from multiple neurons to minimize cell‐specific bias in immunofluorescence‐stained samples. The presence of PAJ component signals at each synapse was assessed by visual inspection using confocal or SIM images. The percentage of synapses showing detectable PAJ component localization was then calculated for each synapse type.

## 3 Results

### 3.1 Four Types of Synapses in Cultured Hippocampal Neurons

**FIGURE 1:**
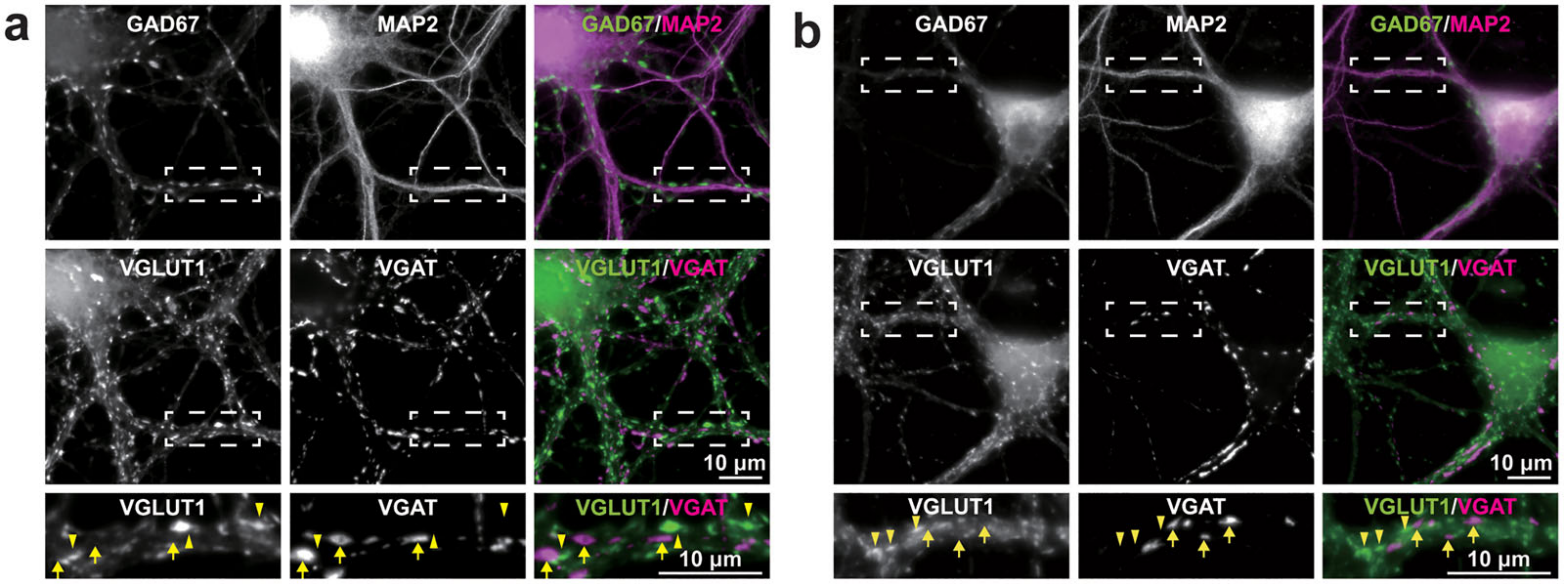
Identification of four types of synapses in cultured hippocampal neurons. Representative immunofluorescence images using the indicated Abs in respective panels. (a) Cultured hippocampal excitatory neurons at 21 DIV. Top row, low‐magnification images of GAD67 and MAP2; middle row, low‐magnification images of VGLUT1 and VGAT; and bottom row, high‐magnification images of the boxed region in the upper panels. Arrowheads, representative *E*→*E* synapses. Arrows, representative *I*→*E* synapses. (b) Cultured inhibitory neurons at 21 DIV. Top row, low‐magnification images of GAD67 and MAP2; middle row, low‐magnification images of VGLUT1 and VGAT; and bottom row, high‐magnification images of the boxed region in the upper panels. Arrowheads, representative *E*→*I* synapses. Arrows, representative *I*→*I* synapses. Images are representative of three independent experiments.

To identify the four types of synapses in cultured mouse hippocampal neurons at 21 days in vitro (DIV), we utilized our previously established method (Komaki et al. 2025). The GAD67‐negative and GAD67‐positive neurons were identified as excitatory neurons and inhibitory neurons, respectively (Figure 1a,b). The MAP2‐positive processes were identified as dendrites. Using this method to distinguish between excitatory and inhibitory neurons and to identify dendrites, VGLUT1‐positive puncta and VGAT‐positive puncta observed on the dendrites of excitatory neurons were identified as *E*→*E* synapses (arrowheads) and *I*→*E* synapses (arrows), respectively (Figure 1a). Similarly, those observed on the dendrites of inhibitory neurons were identified as *E*→*I* synapses (arrowheads) and *I*→*I* synapses (arrows), respectively (Figure 1b). Thus, the four types of synapses were identified in cultured mouse hippocampal neurons.

### 3.2 Localization of PAJ Components at all Four Types of Synapses in Cultured Hippocampal Neurons

**FIGURE 2:**
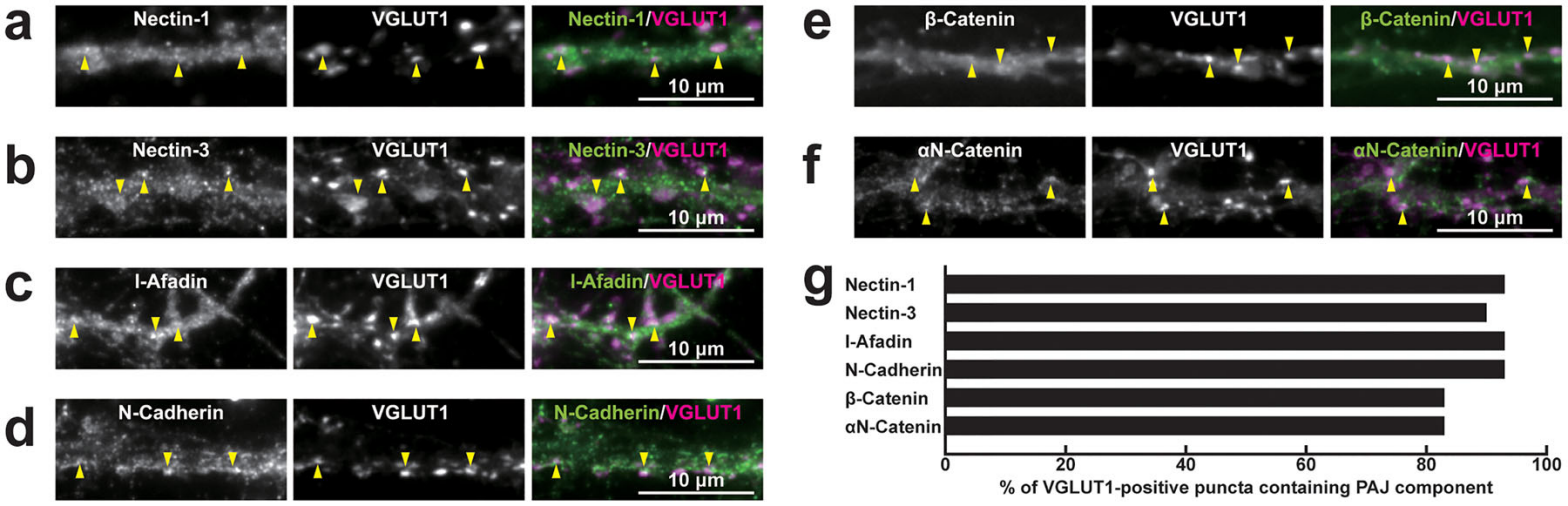
Localization of Puncta adherentia junction (PAJ) components at *E*→*E* synapses in cultured hippocampal neurons at 21 DIV. Representative immunofluorescence images using the indicated Abs in respective panels. (a–f) Arrowheads, representative *E*→*E* synapses showing colocalization of the PAJ component signals and VGLUT1 signals. (g) Quantification of the proportion of *E*→*E* synapses in which the indicated PAJ components were detected in close proximity to VGLUT1‐positive puncta on the dendrites of excitatory neurons. Bars represent the percentage of VGLUT1‐positive puncta with adjacent signals for nectin‐1, nectin‐3, l‐afadin, N‐cadherin, β‐catenin, and αN‐catenin. Images are representative of three independent experiments.

**FIGURE 3:**
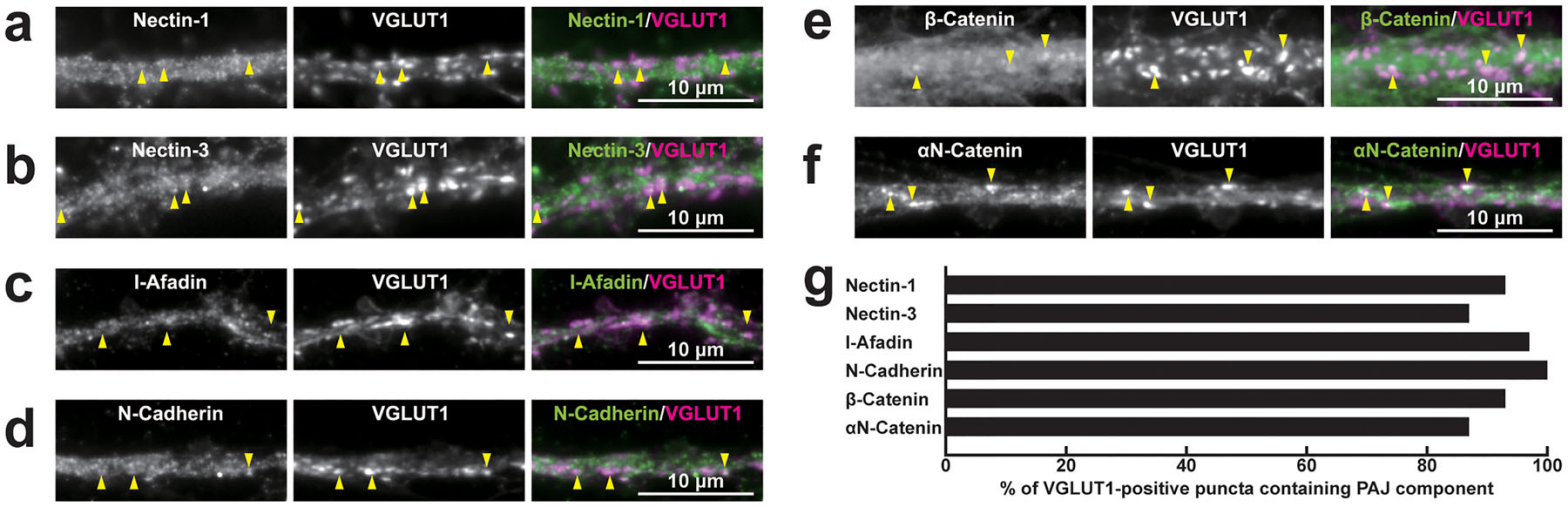
Localization of PAJ components at *E*→*I* synapses in cultured hippocampal neurons at 21 DIV. Representative immunofluorescence images using the indicated Abs in respective panels. (a–f) Arrowheads, representative *E*→*I* synapses showing colocalization of the PAJ component signals and VGLUT1 signals. (g) Quantification of the proportion of *E*→*I* synapses in which the indicated PAJ components were detected in close proximity to VGLUT1‐positive puncta on the dendrites of inhibitory neurons. Bars represent the percentage of VGLUT1‐positive puncta with adjacent signals for nectin‐1, nectin‐3, l‐afadin, N‐cadherin, β‐catenin, and αN‐catenin. Images are representative of three independent experiments.

**FIGURE 4:**
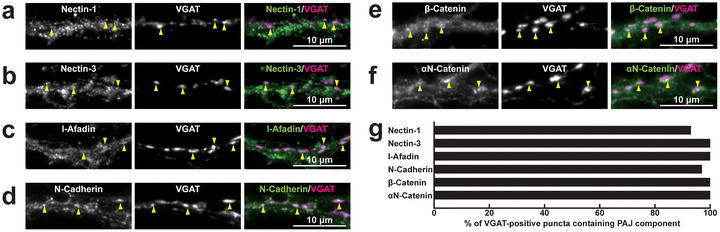
Localization of PAJ components at *I*→*E* synapses in cultured hippocampal neurons at 21 DIV. Representative immunofluorescence images using the indicated Abs in respective panels. (a–f) Arrowheads, representative *I*→*E* synapses showing colocalization of the PAJ component signals and VGAT signals. (g) Quantification of the proportion of *I*→*E* synapses in which the indicated PAJ components were detected in close proximity to VGAT‐positive puncta on the dendrites of excitatory neurons. Bars represent the percentage of VGAT‐positive puncta with adjacent signals for nectin‐1, nectin‐3, l‐afadin, N‐cadherin, β‐catenin, and αN‐catenin. Images are representative of three independent experiments.

**FIGURE 5:**
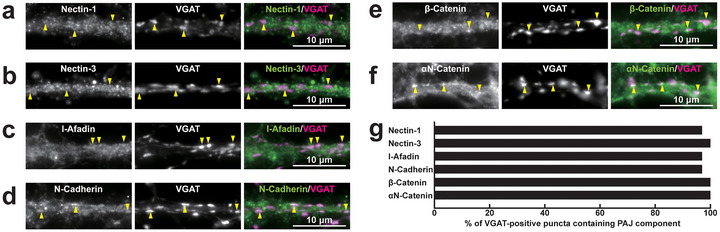
Localization of PAJ components at *I*→*I* synapses in cultured hippocampal neurons at 21 DIV. Representative immunofluorescence images using the indicated Abs in respective panels. (a–f) Arrowheads, representative *I*→*I* synapses showing colocalization of the PAJ component signals and VGAT signals. (g) Quantification of the proportion of *I*→*I* synapses in which the indicated PAJ components were detected in close proximity to VGAT‐positive puncta on the dendrites of inhibitory neurons. Bars represent the percentage of VGAT‐positive puncta with adjacent signals for nectin‐1, nectin‐3, l‐afadin, N‐cadherin, β‐catenin, and αN‐catenin. Images are representative of three independent experiments.

**FIGURE 6:**
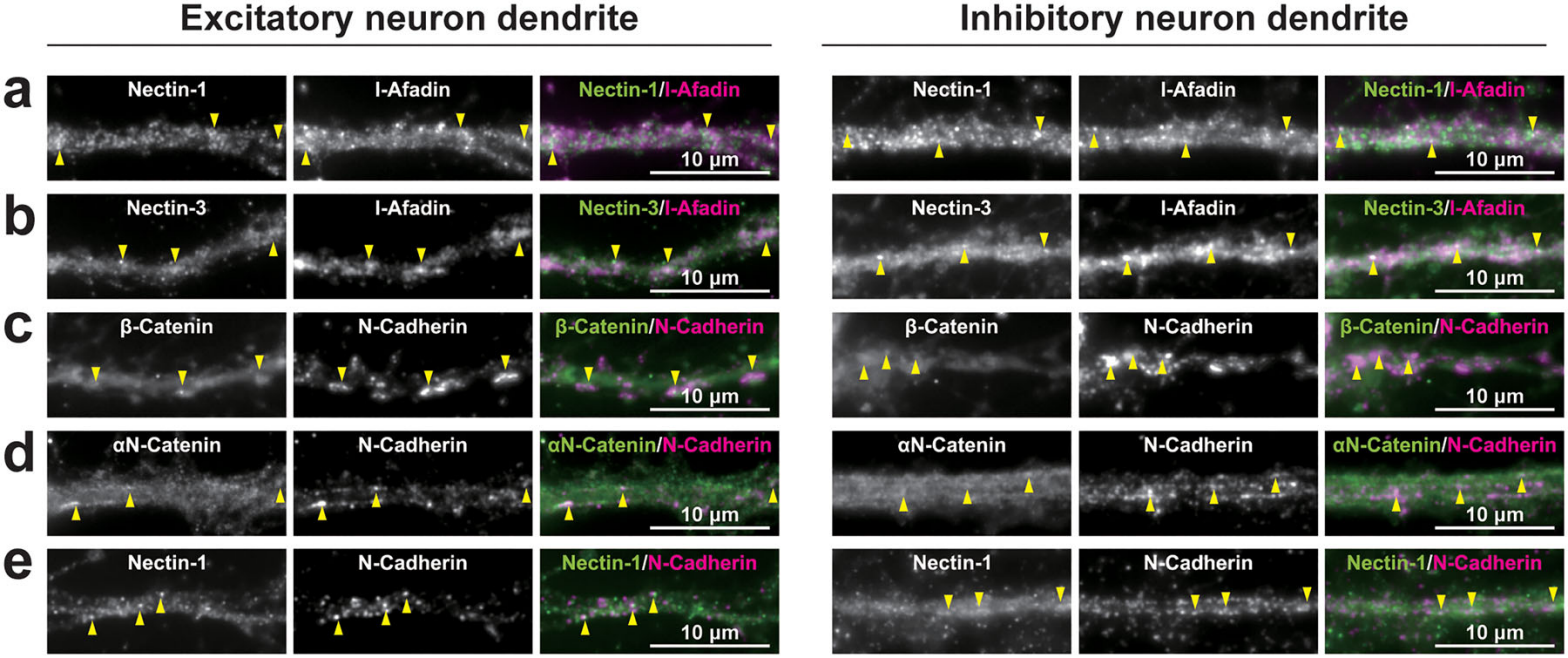
Colocalization of PAJ components in cultured hippocampal neurons at 21 DIV. Representative immunofluorescence images using the indicated Abs in respective panels. Arrowheads, representative puncta of the indicated PAJ component signals along the dendrites of excitatory or inhibitory neurons. Images are representative of three independent experiments.

To investigate the localization of PAJ components at different types of synapses, we examined their presence in cultured mouse hippocampal neurons at 21 DIV. Immunofluorescence signals for nectin‐1, nectin‐3, l‐afadin, N‐cadherin, β‐catenin, and αN‐catenin were all observed in close proximity to presynaptic markers at all four types of synapses: near the VGLUT1 signal on excitatory neurons (Figure 2a–f) and inhibitory neurons (Figure 3a–f), as well as near the VGAT signal on excitatory neurons (Figure 4a–f) and inhibitory neurons (Figure 5a–f). Quantitative analysis showed that these PAJ components were detected in a substantial proportion of *E*→*E*, *E*→*I*, *I*→*E*, and *I*→*I* synapses (Figures 2g, 3g, 4g, and 5g). Double immunofluorescence staining further revealed that each pair of PAJ components exhibited substantial colocalization: nectin‐1 and l‐afadin (Figure 6a), nectin‐3 and l‐afadin (Figure 6b), β‐catenin and N‐cadherin (Figure 6c), αN‐catenin and N‐cadherin (Figure 6d), and nectin‐1 and N‐cadherin (Figure 6e). These results indicate that nectin‐1, nectin‐3, l‐afadin, N‐cadherin, β‐catenin, and αN‐catenin are colocalized at all four types of synapses in cultured hippocampal neurons (Table 2).

**TABLE 2:** Proportion of synapses showing localization of PAJ components.

		*E*→*E* synapses	*E*→*I* synapses	*I*→*E* synapses	*I*→*I* synapses
**In vitro**	**Cultured hippocampal neurons**	Nectin‐1 93% Nectin‐3 90% l‐Afadin 93% N‐Cadherin 93% β‐Catenin 83% αN‐Catenin 83%	Nectin‐1 93% Nectin‐3 87% l‐Afadin 97% N‐Cadherin 100% β‐Catenin 93% αN‐Catenin 87%	Nectin‐1 93% Nectin‐3 100% l‐Afadin 100% N‐Cadherin 97% β‐Catenin 100% αN‐Catenin 100%	Nectin‐1 97% Nectin‐3 100% l‐Afadin 97% N‐Cadherin 97% β‐Catenin 100% αN‐Catenin 100%
**In vivo**	**CA1**	Nectin‐1 60% Nectin‐3 50% l‐Afadin 70% N‐Cadherin 67% β‐Catenin 70% αN‐Catenin 77%	Nectin‐1 17% Nectin‐3 50% l‐Afadin 23% N‐Cadherin 53% β‐Catenin 60% αN‐Catenin 63%	Nectin‐1 20% Nectin‐3 20% l‐Afadin 67% N‐Cadherin 73% β‐Catenin 70% αN‐Catenin 77%	Nectin‐1 47% Nectin‐3 50% l‐Afadin 57% N‐Cadherin 70% β‐Catenin 57% αN‐Catenin 80%
	**DG**	Nectin‐1 23% Nectin‐3 23% l‐Afadin 27% N‐Cadherin 77% β‐Catenin 77% αN‐Catenin 83%	Nectin‐1 0% N‐Cadherin 20%	Nectin‐1 0% N‐Cadherin 80%	Nectin‐1 0% N‐Cadherin 40%
	**CA3**	Nectin‐1 97% N‐Cadherin 90%	Nectin‐1 0% N‐Cadherin 17%	Nectin‐1 0% N‐Cadherin 83%	Nectin‐1 0% N‐Cadherin 30%

*Note:* Values indicate the percentage of synapses positive for each molecule. Molecules not listed in the table were not examined in this study.

### 3.3 Extrasynaptic Localization of the PAJ Components at Four Types of Synapses in Cultured Hippocampal Neurons

**FIGURE 7:**
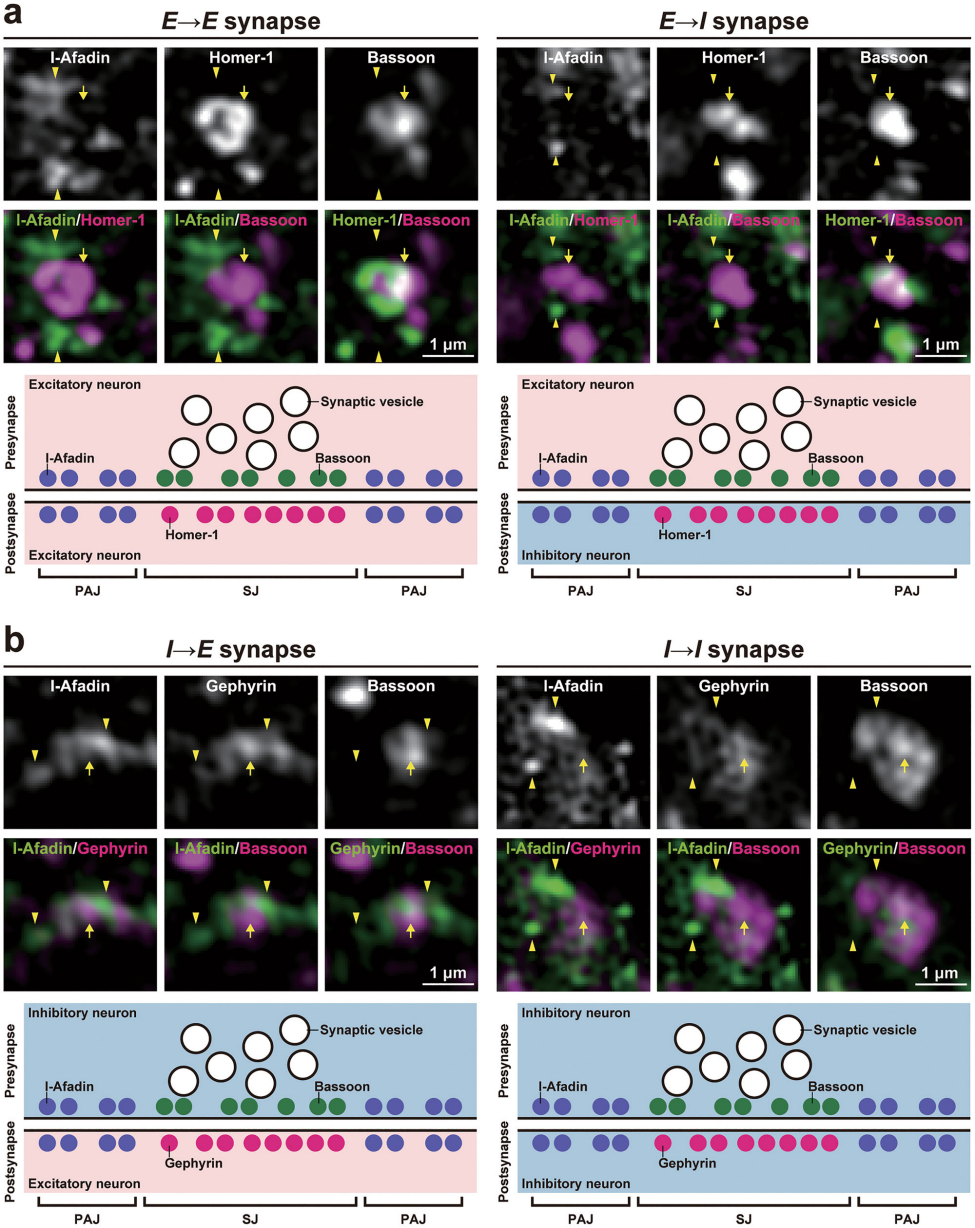
Extrasynaptic localization of the PAJs at four types of synapses in cultured hippocampal neurons at 21 DIV. Representative structured illumination microscopic (SIM) images using the indicated Abs in respective panels. (a) SIM images of excitatory synapses. (b) SIM images of inhibitory synapses. Arrowheads, localization of the l‐afadin signals along the dendrites of excitatory or inhibitory neurons, as identified in Figure 1. Arrows, localization of the indicated pre‐ or postsynaptic marker signals at each synapse. The schematic diagrams shown below the SIM images illustrate the relative distribution of l‐afadin (a PAJ component) and synaptic junction (SJ) markers at *E*→*E*, *E*→*I*, *I*→*E*, and *I*→*I* synapses. In each schematic, synaptic vesicles are clustered at the presynaptic active zone, bassoon is localized along the presynaptic SJ, and homer‐1 or gephyrin marks the postsynaptic SJ of excitatory or inhibitory synapses, respectively. In contrast, l‐afadin is positioned at extrasynaptic membrane regions flanking the SJ on both pre‐ and postsynaptic sides. This distribution is consistent with its localization at PAJs across all four synapse types. Images are representative of three independent experiments.

Previous electron microscopy studies have shown that the PAJs localize at the extrasynaptic region and connect axon terminals and dendritic shafts at *E*→*E* synapses (Mizoguchi et al. 2002; Uchida et al. 1996). To further investigate the localization of PAJ components relative to SJs, we compared the localizations of the PAJs and the SJs in cultured hippocampal neurons using super‐resolution structured illumination microscopy (SIM). To determine the localization of the SJs at four types of synapses, bassoon, homer‐1, and gephyrin were used as markers because bassoon and homer‐1 are associated with the SJs at the presynaptic and postsynaptic sides of excitatory synapses, respectively (Ehrengruber et al. 2004; tom Dieck et al. 1998), whereas bassoon and gephyrin are associated with the SJs at the presynaptic and postsynaptic sides of inhibitory synapses, respectively (Groeneweg et al. 2018; Richter et al. 1999). In higher‐magnification images by SIM, the immunofluorescence signal for l‐afadin, representing the PAJ components, localized near, but did not colocalize with, the signals for bassoon and homer‐1 at *E*→*E* and *E*→*I* synapses (Figure 7a). Similarly, the signal for l‐afadin localized near, but did not colocalize with, the signals for bassoon and gephyrin at *I*→*E* and *I*→*I* synapses (Figure 7b). These results indicate that the PAJ components localize at the extrasynaptic region and likely connect axon terminals and dendritic shafts at the extrasynaptic region of all four types of synapses in cultured hippocampal neurons.

### 3.4 Localization of PAJ Components at *E*→*E* Synapses and a Subset of *I*→*I* Synapses in the Hippocampus

**FIGURE 8:**
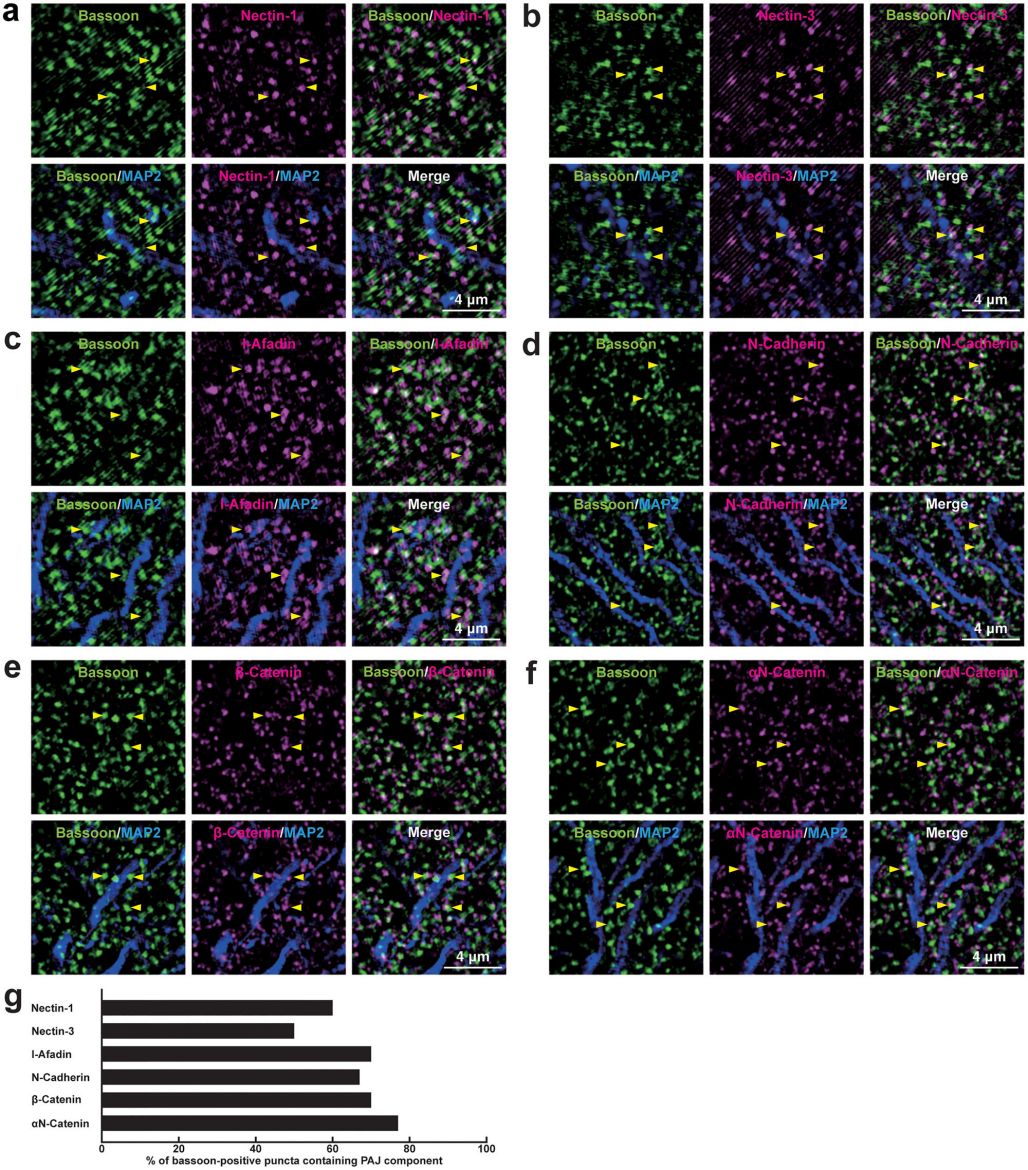
Localization of the PAJs at *E*→*E* synapses in the CA1 of the hippocampus in vivo. (a–f) Representative SIM images of the *stratum radiatum* of the CA1 in P56 mouse hippocampi using the indicated Abs in respective panels. Arrowheads, representative synapses showing colocalization of the bassoon signals with PAJ component signals. (g) Quantification of the proportion of *E*→*E* synapses in which the indicated PAJ components were detected in close proximity to bassoon‐positive puncta on the MAP2‐positive dendrites of excitatory neurons. Bars represent the percentage of bassoon‐positive puncta with adjacent signals for nectin‐1, nectin‐3, l‐afadin, N‐cadherin, β‐catenin, and αN‐catenin. Images are representative of three independent experiments.

**FIGURE 9:**
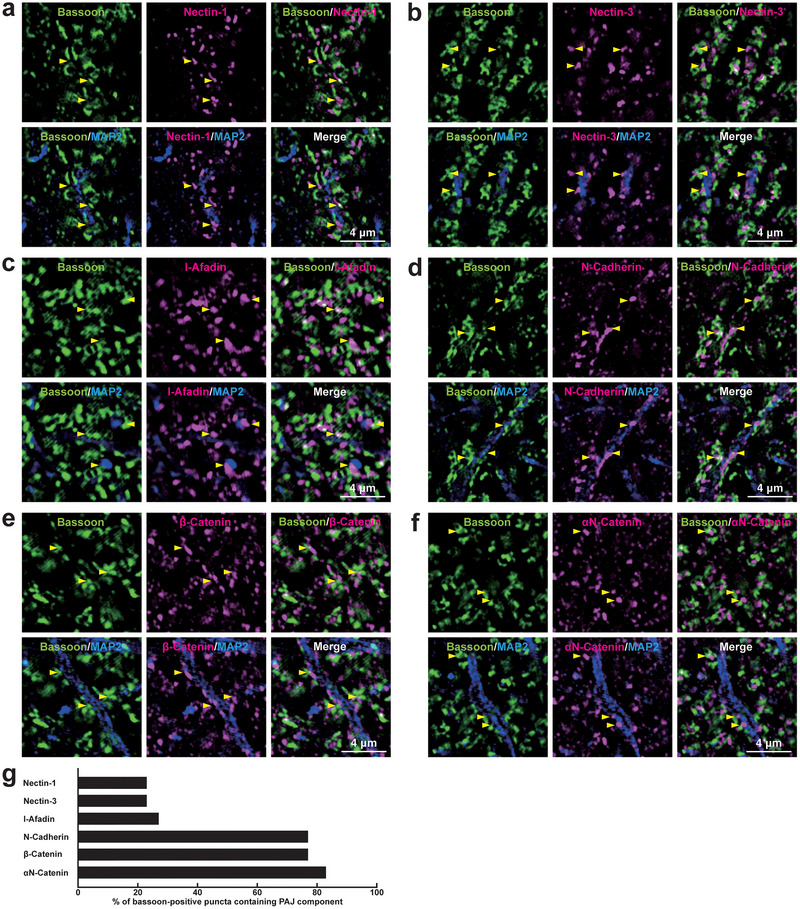
Localization of the PAJs at *E*→*E* synapses in the DG of the hippocampus in vivo. (a–f) Representative SIM images of the *stratum polymorphum* of the DG in P56 mouse hippocampi using the indicated Abs in respective panels. Arrowheads, representative synapses showing colocalization of the bassoon signals with the indicated PAJ component signals. (g) Quantification of the proportion of *E*→*E* synapses in which the indicated PAJ components were detected in close proximity to bassoon‐positive puncta on the MAP2‐positive dendrites of excitatory neurons. Bars represent the percentage of bassoon‐positive puncta with adjacent signals for nectin‐1, nectin‐3, l‐afadin, N‐cadherin, β‐catenin, and αN‐catenin. Images are representative of three independent experiments.

**FIGURE 10:**
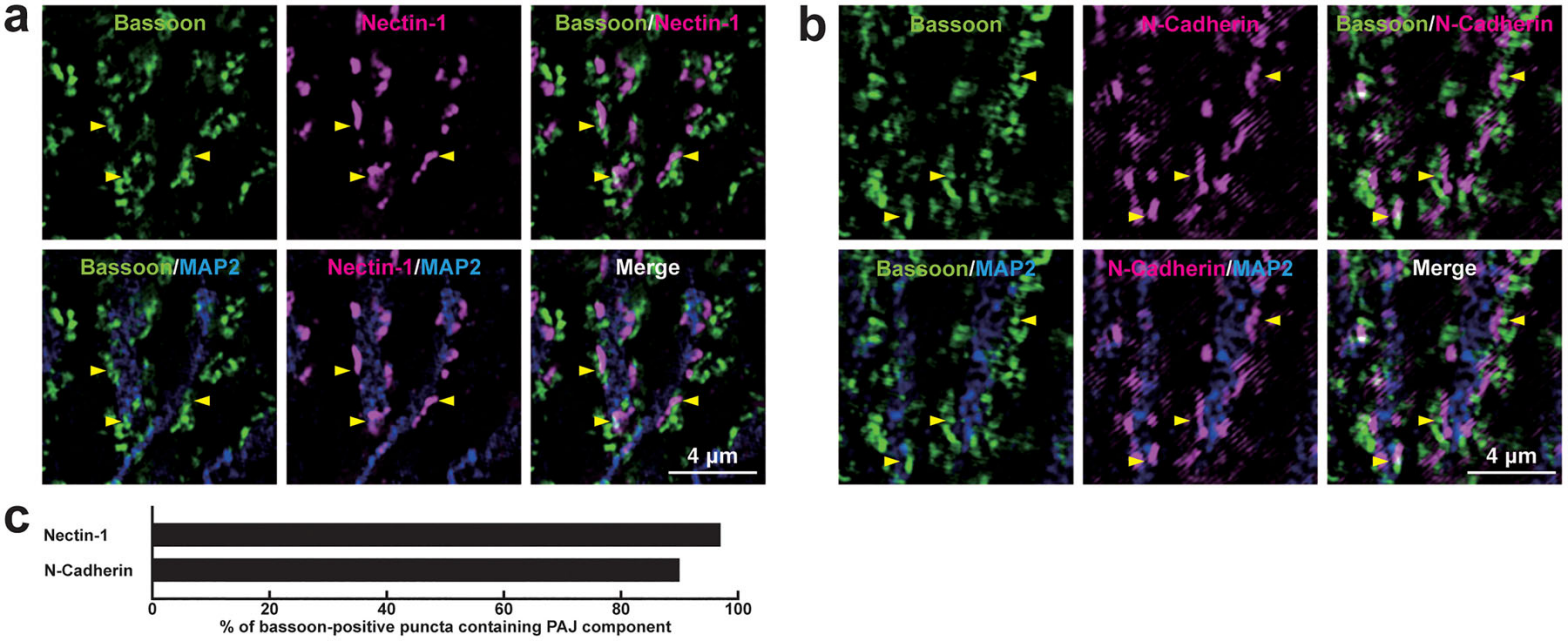
Localization of the PAJs at *E*→*E* synapses in the CA3 of the hippocampus in vivo. (a, b) Representative SIM images of the *stratum lucidum* of the CA3 in P56 mouse hippocampi using the indicated Abs in respective panels. Arrowheads, representative synapses showing colocalization of the bassoon signals with the indicated PAJ component signals. (c) Quantification of the proportion of *E*→*E* synapses in which the indicated PAJ components were detected in close proximity to bassoon‐positive puncta on the MAP2‐positive dendrites of excitatory neurons. Bars represent the percentage of bassoon‐positive puncta with adjacent signals for nectin‐1 and N‐cadherin. Images are representative of three independent experiments.

**FIGURE 11:**
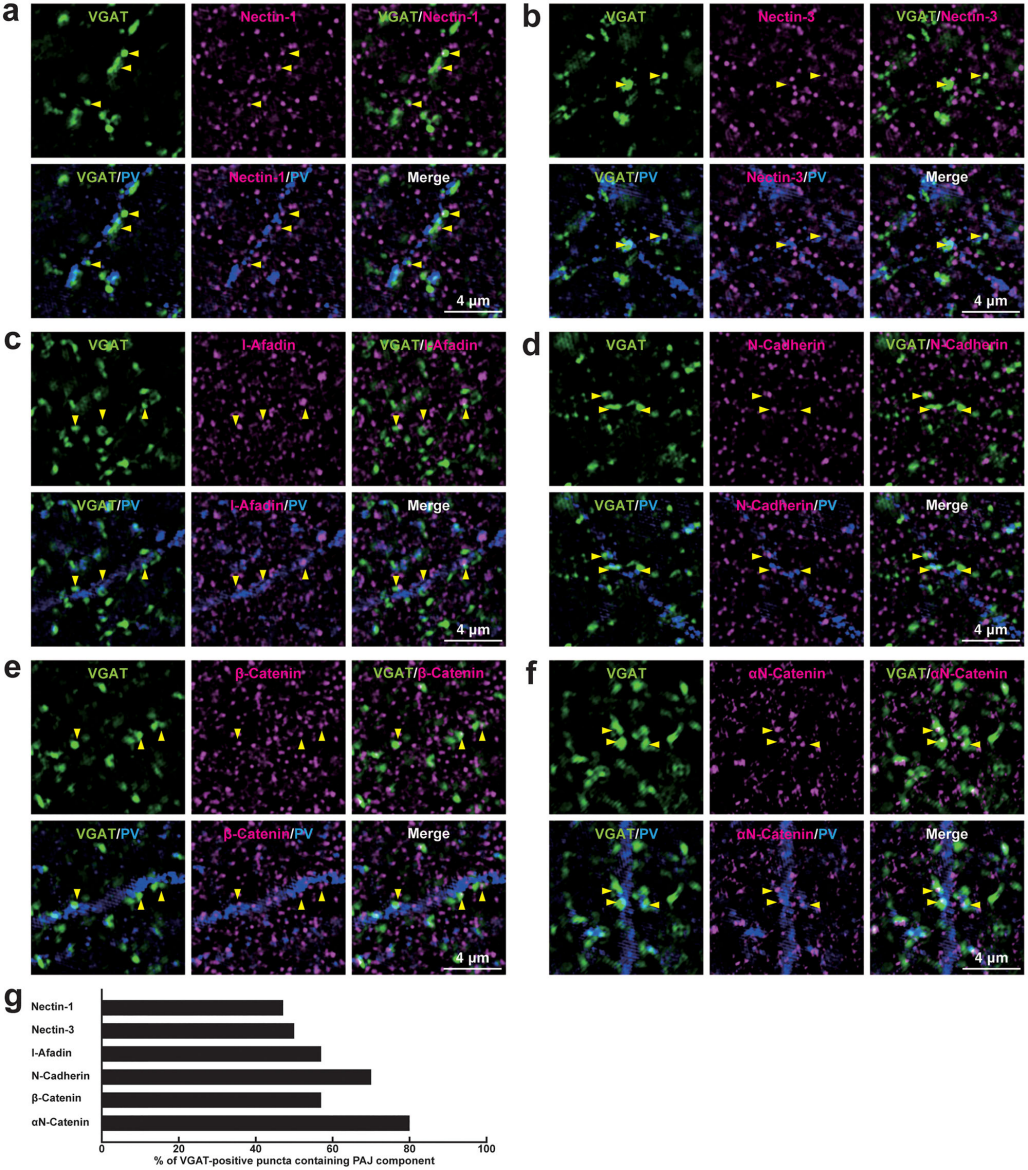
Localization of the PAJs at a subset of *I*→*I* synapses in the CA1 of the hippocampus in vivo. (a–f) Representative SIM images of the *stratum radiatum* of the CA1 in P56 mouse hippocampi using the indicated Abs in respective panels. Arrowheads, representative *I*→*I* synapses showing colocalization of the VGAT signals on PV‐positive inhibitory neurons with the indicated PAJ component signals. (g) Quantification of the proportion of *I*→*I* synapses in which the indicated PAJ components were detected in close proximity to VGAT‐positive puncta on the PV‐positive dendrites of inhibitory neurons. Bars represent the percentage of VGAT‐positive puncta with adjacent signals for nectin‐1, nectin‐3, l‐afadin, N‐cadherin, β‐catenin, and αN‐catenin. Images are representative of three independent experiments.

**FIGURE 12:**
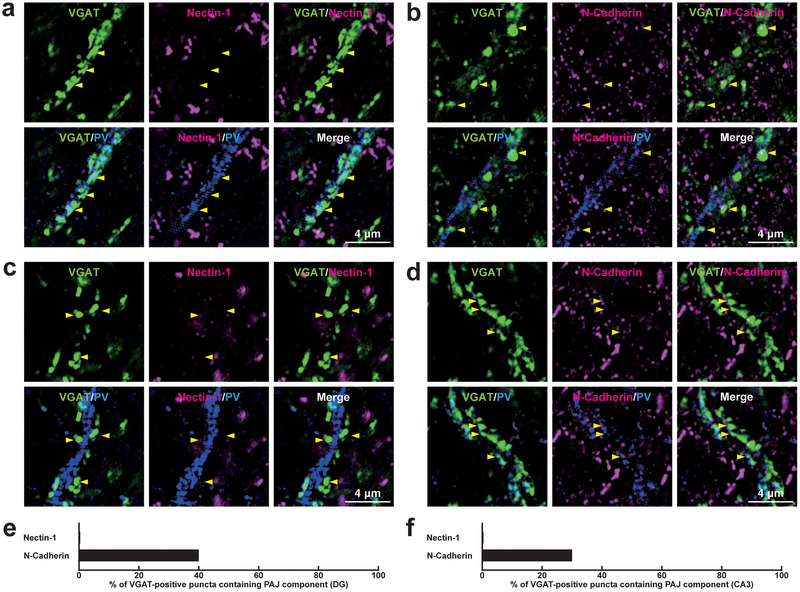
Localization of the PAJ components at *I*→*I* synapses in the DG and the CA3 of the hippocampus in vivo. Representative SIM images of the hippocampus in P56 mice using the indicated Abs in respective panels. (a, b) SIM images of the *stratum polymorph* of the DG. (c, d) SIM images of the *stratum lucidum* of the CA3. Arrowheads, representative VGAT‐positive *I*→*I* synapses on PV‐positive inhibitory neurons. (e, f) Quantification of the proportion of *I*→*I* synapses in which the indicated PAJ components were detected in close proximity to VGAT‐positive puncta on the PV‐positive dendrites of inhibitory neurons: DG (e) and CA3 (f). Bars represent the percentage of VGAT‐positive puncta with adjacent signals for nectin‐1 and N‐cadherin. Images are representative of three independent experiments.

**FIGURE 13:**
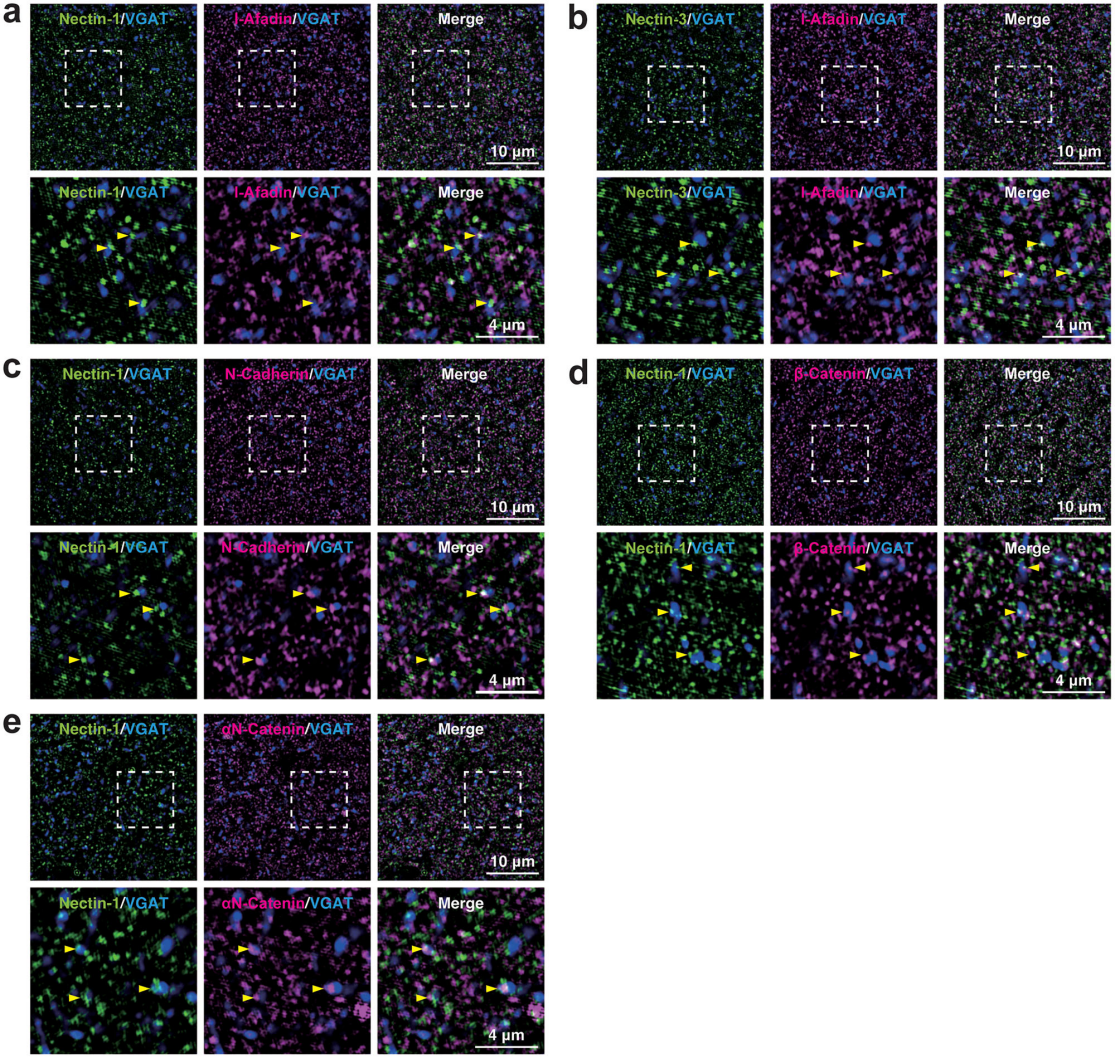
Colocalization of the PAJ components at a subset of *I*→*I* synapses in the CA1 of the hippocampus in vivo. Representative SIM images of the hippocampus in P56 mice using the indicated Abs in respective panels. (a–e) SIM images of the *stratum radiatum* of the CA1. The boxed region in the upper panel is shown at higher magnification in the lower panel. Arrowheads, the puncta with the indicated PAJ component signals. Images are representative of three independent experiments.

We then examined whether PAJ components are localized not only at *E*→*E* synapses but also at other types of synapses in the mouse hippocampus. Immunofluorescence signals for PAJ components, such as nectin‐1, nectin‐3, l‐afadin, N‐cadherin, β‐catenin, and αN‐catenin, were observed at *E*→*E* synapses formed between Schaffer collateral axon terminals and the dendrites of pyramidal cells in the *stratum radiatum* of the CA1 (Figure 8a–g; Table 2). Immunofluorescence signals were also observed at *E*→*E* synapses formed between mossy fiber axon terminals and the dendrites of the mossy cells in the *stratum polymorphum* of the DG, although nectin‐1, nectin‐3, and l‐afadin were observed at comparatively lower proportions (Figure 9a–g; Table 2). The signals for nectin‐1 and N‐cadherin, representing the PAJ components, were also observed at *E*→*E* synapses that were formed between mossy fiber axon terminals and the dendrites of pyramidal cells in the *stratum lucidum* of the CA3 (Figure 10a–c; Table 2), consistent with previous observations (Majima et al. 2009; Mizoguchi et al. 2002; Nishioka et al. 2000). In addition to these *E*→*E* synapses, the signals for the PAJ components, such as nectin‐1, nectin‐3, l‐afadin, N‐cadherin, β‐catenin, and αN‐catenin, were each observed at a subset of *I*→*I* synapses formed between axon terminals of unidentified inhibitory neurons and dendrites of PV‐positive inhibitory neurons, presumably basket cells, in the *stratum radiatum* of the CA1 (Figure 11a–g; Table 2), whereas the remaining synapses did not exhibit significant labeling for these molecules. The signals for N‐cadherin, but not for nectin‐1, were localized at a subset of *I*→*I* synapses in the DG and CA3 (Figure 12a–f; Table 2). In the CA1, the signals for the PAJ components colocalized with those for VGAT and nectins, as demonstrated by following marker combinations: nectin‐1 or nectin‐3 with l‐afadin (Figure 13a, b); nectin‐1 with N‐cadherin (Figure 13c), β‐catenin (Figure 13d), or αN‐catenin (Figure 13e). These results demonstrate that PAJ components are localized not only at *E*→*E* synapses but also at a subset of *I*→*I* synapses, particularly in the CA1, suggesting the existence of PAJ‐like adhesive structures at specific inhibitory synapses.

### 3.5 Localization of Specific PAJ Components at *E*→*I* Synapses and *I*→*E* Synapses in the Hippocampus

**FIGURE 14:**
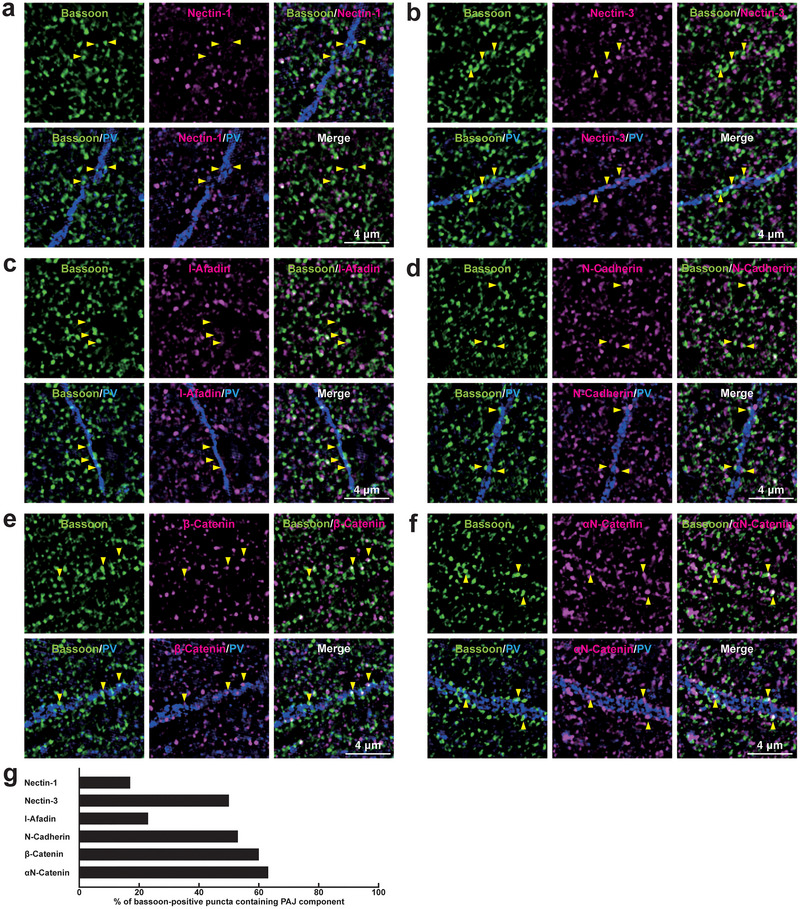
Localization of the PAJ components at *E*→*I* synapses in the CA1 of the hippocampus in vivo. (a–f) Representative SIM images of the *stratum radiatum* of the CA1 in P56 mouse hippocampi using the indicated Abs in respective panels. Arrowheads, representative bassoon‐positive *E*→*I* synapses on PV‐positive inhibitory neurons. (g) Quantification of the proportion of *E*→*I* synapses in which the indicated PAJ components were detected in close proximity to bassoon‐positive puncta on the PV‐positive dendrites of inhibitory neurons. Bars represent the percentage of bassoon‐positive puncta with adjacent signals for nectin‐1, nectin‐3, l‐afadin, N‐cadherin, β‐catenin, and αN‐catenin. Images are representative of three independent experiments.

**FIGURE 15:**
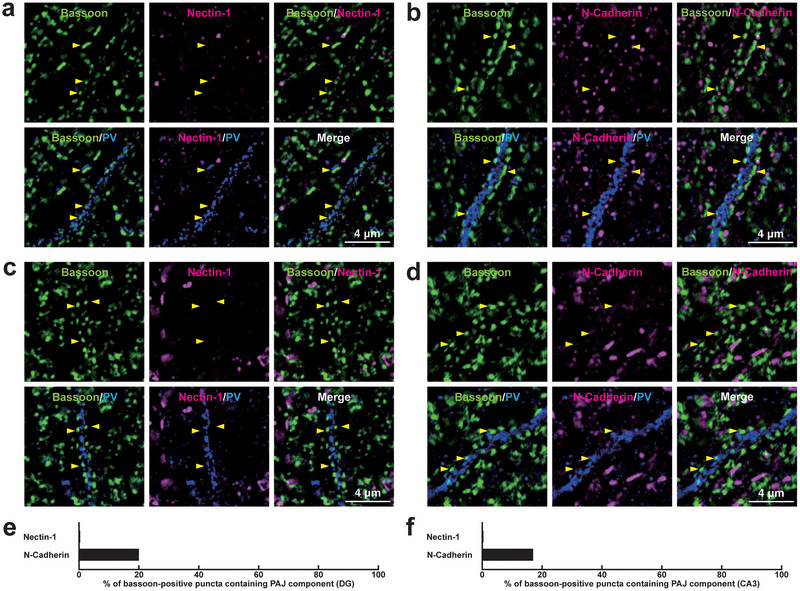
Localization of the PAJ components at *E*→*I* synapses in the DG and the CA3 of the hippocampus in vivo. Representative SIM images of the hippocampus in P56 mice using the indicated Abs in respective panels. (a, b) SIM images of the *stratum polymorph* of the DG. (c, d) SIM images of the *stratum lucidum* of the CA3. Arrowheads, representative *E*→*I* synapses showing the bassoon signals on PV‐positive inhibitory neurons. (e, f) Quantification of the proportion of *E*→*I* synapses in which the indicated PAJ components were detected in close proximity to bassoon‐positive puncta on the PV‐positive dendrites of inhibitory neurons: DG (e) and CA3 (f). Bars represent the percentage of VGAT‐positive puncta with adjacent signals for nectin‐1 and N‐cadherin. Images are representative of three independent experiments.

We finally investigated whether the PAJs are also formed at the remaining two synapses: *E*→*I* and *I*→*E* synapses. At *E*→*I* synapses formed between Schaffer collateral axon terminals and the dendrites of basket cells in the *stratum radiatum* of the CA1, the immunofluorescence signals were observed for nectin‐3, N‐cadherin, β‐catenin, and αN‐catenin, whereas the signals for nectin‐1 and l‐afadin were occasionally observed (Figure 14a–g; Table 2). Neither the signals for nectin‐1 nor N‐cadherin were observed at appreciable levels at *E*→*I* synapses formed between mossy fiber axon terminals and the dendrites of basket cells in the *stratum polymorphum* of the DG, where N‐cadherin was observed only in a minority of synapses (Figure 15a,b,e; Table 2). Similarly, at *E*→*I* synapses formed between mossy fiber axon terminals and the dendrites of basket cells in the *stratum lucidum* of the CA3, nectin‐1 was not observed, and N‐cadherin was occasionally observed (Figure 15c,d,f; Table 2).

**FIGURE 16:**
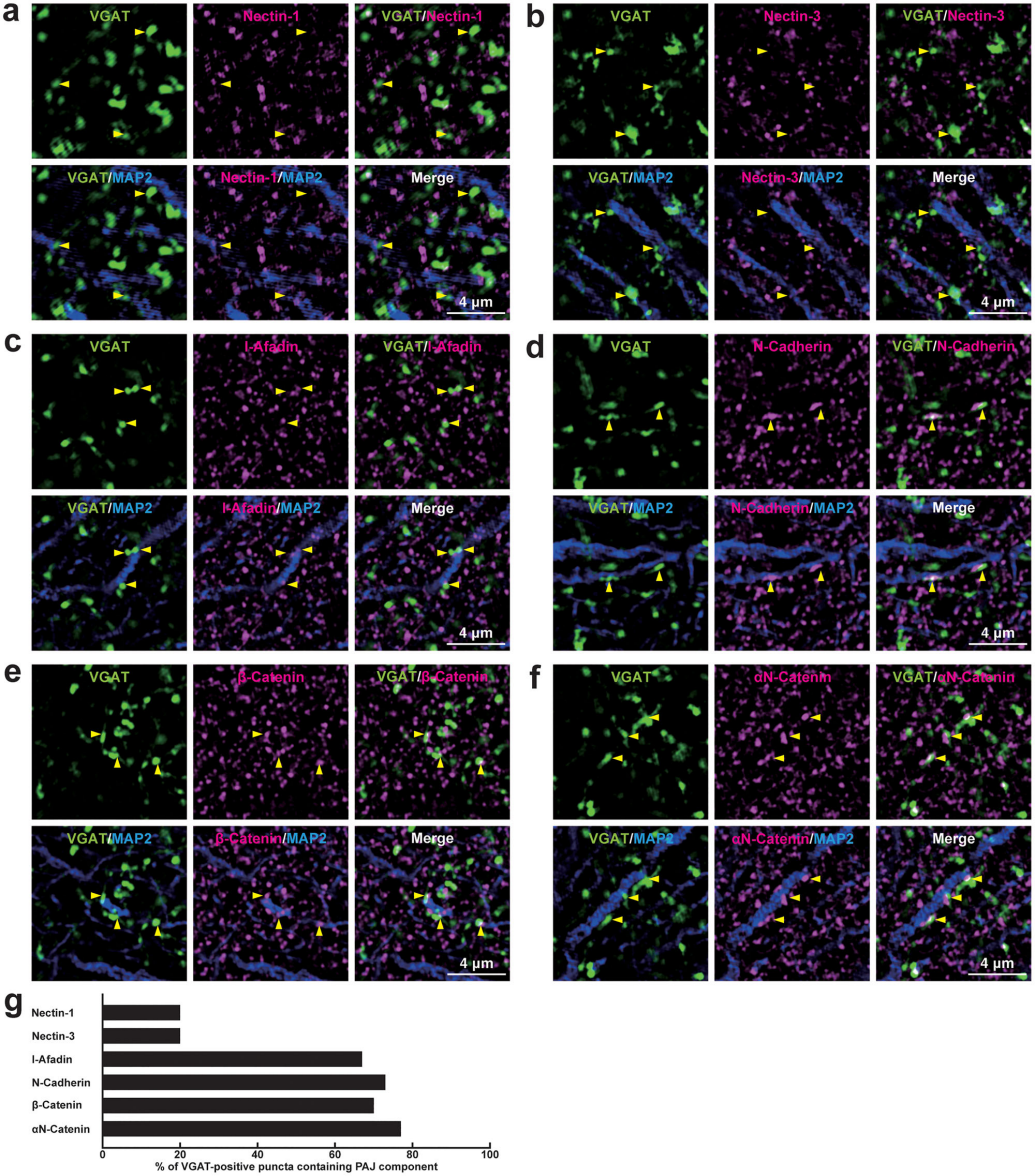
Localization of the PAJ components at *I*→*E* synapses in the CA1 of the hippocampus in vivo. (a–f) Representative SIM images of the *stratum radiatum* of the CA1 in P56 mouse hippocampi using the indicated Abs in respective panels. Arrowheads, representative VGAT‐positive *I*→*E* synapses on MAP2‐positive neurons. (g) Quantification of the proportion of *I*→*E* synapses in which the indicated PAJ components were detected in close proximity to VGAT‐positive puncta on the MAP2‐positive dendrites of excitatory neurons. Bars represent the percentage of VGAT‐positive puncta with adjacent signals for nectin‐1, nectin‐3, l‐afadin, N‐cadherin, β‐catenin, and αN‐catenin. Images are representative of three independent experiments.

**FIGURE 17:**
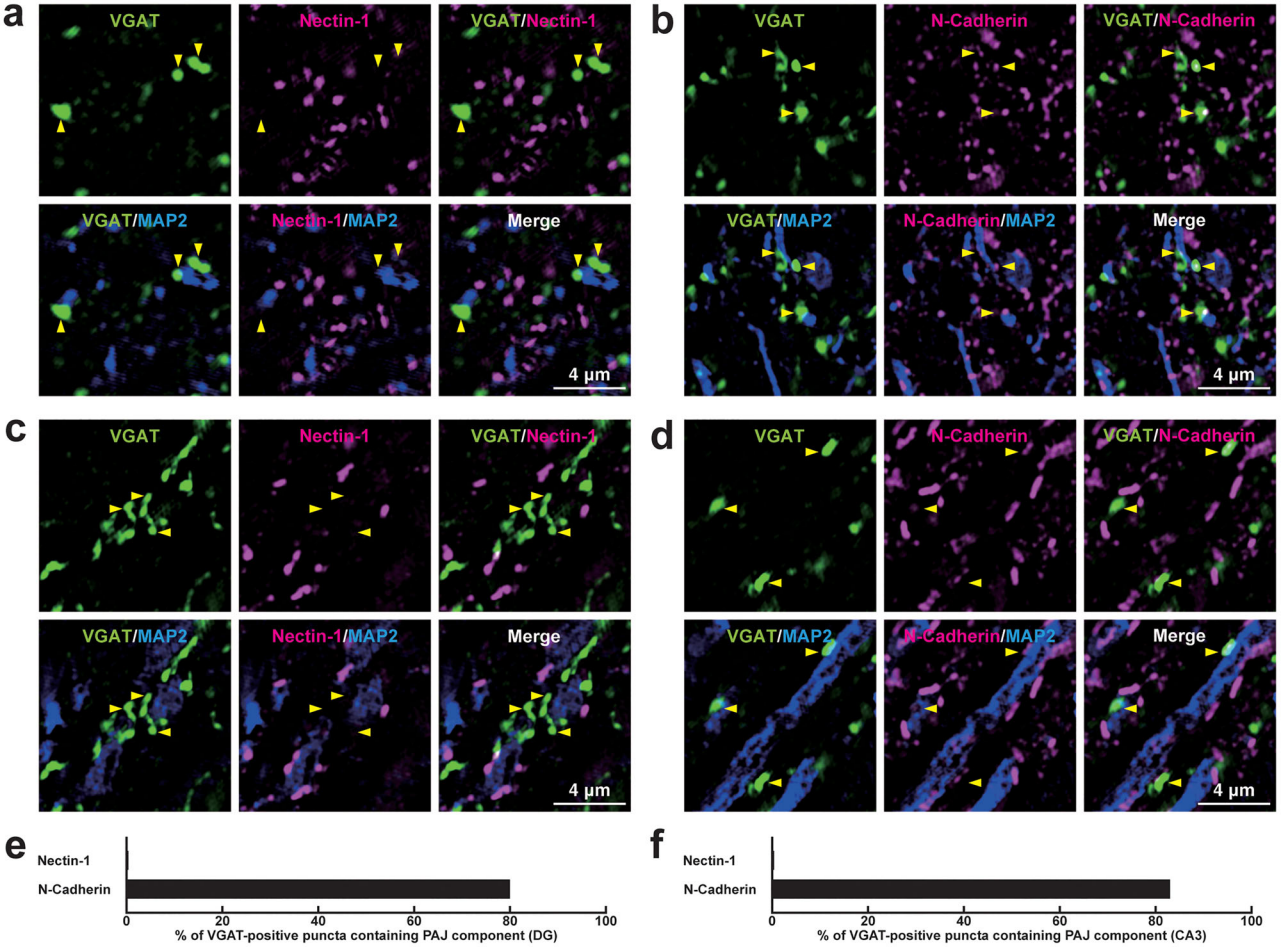
Localization of the PAJ components at *I*→*E* synapses in the DG and the CA3 of the hippocampus in vivo. Representative SIM images of the hippocampus in P56 mice using the indicated Abs in respective panels. (a, b) SIM images of the *stratum polymorph* of the DG. (c, d) SIM images of the *stratum lucidum* of the CA3. Arrowheads, representative *I*→*E* synapses showing the VGAT signals on MAP2‐positive neurons. (e, f) Quantification of the proportion of *I*→*E* synapses in which the indicated PAJ components were detected in close proximity to VGAT‐positive puncta on the MAP2‐positive dendrites of excitatory neurons: DG (e) and CA3 (f). Bars represent the percentage of VGAT‐positive puncta with adjacent signals for nectin‐1 and N‐cadherin. Images are representative of three independent experiments.

At *I*→*E* synapses formed between the axon terminals of unidentified inhibitory neurons and the somata of pyramidal cells at the *stratum pyramidale* of the CA1, the signals were observed for l‐afadin, N‐cadherin, αN‐catenin, and β‐catenin, whereas the signals for nectin‐1 and nectin‐3 were observed in only a minority of synapses (Figure 16a–g; Table 2). The signal for N‐cadherin but not nectin‐1 was observed at *I*→*E* synapses formed between axon terminals of unidentified inhibitory neurons and the dendrites of mossy cells in the *stratum polymorphum* of the DG (Figure 17a,b,e; Table 2), and between axon terminals of unidentified inhibitory neurons and the dendrites of pyramidal cells in the *stratum lucidum* of the CA3 (Figure 17c,d,f; Table 2).

## 4 Discussion

Previous studies have demonstrated the presence of PAJs at *E*→*E* synapses in the hippocampus. In the present study, we found that PAJ components, including nectin‐1, nectin‐3, l‐afadin, N‐cadherin, β‐catenin, and αN‐catenin, were observed not only at the extrasynaptic region of *E*→*E* synapses but also at *E*→*I*, *I*→*E*, and *I*→*I* synapses in cultured mouse hippocampal neurons. We further examined PAJs in adult mouse hippocampus and found their presence at *E*→*E* synapses in the DG as well as in the CA1 and the CA3. Additionally, we observed PAJs at a subset of *I*→*I* synapses on PV‐positive inhibitory neurons in the CA1. In contrast, the signals for nectin‐1 were only occasionally observed at *E*→*I* and *I*→*E* synapses, being detected in a minority of synapses in the CA1. These results highlight the synapse‐type‐specific distribution of PAJ components, suggesting structural diversity in adhesive junctions across different synapses.

PAJs are unique adhesion structures composed of well‐characterized CAMs. Consistent with previous studies (Mizoguchi et al. 2002; Uchida et al. 1996), we confirmed their localization at *E*→*E* synapses in the CA1 and the CA3 and additionally observed them in the DG. Although our analysis did not cover all hippocampal subregions, the observations of PAJs at multiple *E*→*E* synapses in the CA1, the CA3, and the DG suggest that these structures are broadly distributed across the hippocampus.

In the present study, we also found PAJs at a subset of *I*→*I* synapses on PV‐positive inhibitory neurons in the CA1. PV‐positive inhibitory neurons are a major class of fast‐spiking inhibitory neurons (Pelkey et al. 2017). They are essential for synchronizing network activity and maintaining E/I balance in the hippocampus, as they provide precise feedforward and feedback inhibition and are necessary for the generation of network oscillations, such as gamma rhythms (Whittington et al. 1995). These neurons are particularly important for timing‐dependent processes such as oscillatory coordination and information gating (Gonzalez‐Burgos et al. 2010). The observations that PAJs are present at a subset of *I*→*I* synapses on these inhibitory neurons raise the possibility that PAJs may contribute to the selective regulation of inhibitory signaling. Given that PV‐positive inhibitory neurons are highly active and form powerful inhibitory inputs via perisomatic synapses (Pelkey et al. 2017), the presence of PAJs might support the mechanical stability of selected recurrent inhibitory synapses under high‐frequency firing conditions. Further studies will be required to clarify the specific structural and functional roles of PAJs at *I*→*I* synapses.

In this study, we observed that several, but not all, PAJ components were detected at *E*→*I* and *I*→*E* synapses in the adult hippocampus, whereas representative PAJ components were detected at these synapses in vitro. One possible explanation is that the formation of PAJs may be restricted under physiological conditions in vivo. Given that astrocytes and microglia are known to regulate synapse formation and remodeling (Cornell et al. 2022; Kim and Chung 2023), it is possible that these glial cells influence the assembly or stability of PAJs in a synapse‐type or context‐dependent manner. Another possibility is that PAJs at *E*→*I* and *I*→*E* synapses are formed only transiently during specific developmental or physiological stages, such as periods of synaptic refinement or circuit remodeling, and become diminished or absent in the adult brain. Our observations in vitro indicate that hippocampal neurons have the intrinsic capacity to form PAJs at *E*→*I* and *I*→*E* synapses, suggesting that their formation is permissive under certain conditions but may be suppressed or destabilized in vivo. Further investigation will be necessary to clarify the molecular and cellular mechanisms that regulate synapse‐type‐specific assembly of PAJ components in vivo. In addition, it is possible that in the adult hippocampus, PAJs at *E*→*I* and *I*→*E* synapses may contain different sets of adhesion molecules that were not examined in the present study. These alternative components might contribute to the formation of PAJs that fulfill similar structural or functional roles at these synapses in vivo.

CAMs localized at SJs and PAJs play critical roles not only in maintaining synaptic structure but also in regulating synaptic plasticity under both physiological and pathological conditions. These molecules contribute to activity‐dependent remodeling throughout life, a process influenced by experience and environmental factors (Forrest et al. 2018; Holtmaat and Svoboda 2009). Disruption of CAM function, particularly during critical developmental periods, has been implicated in impairments of synaptic plasticity and memory formation, thereby increasing vulnerability to various neuropsychiatric disorders (Lupien et al. 2009; Nemeroff 2016; Short and Baram 2019; X. Wang et al. 2018). A well‐characterized example is neuroligin‐2, a synaptic adhesion molecule critical for maintaining inhibitory neurotransmission and the E/I balance (Ali et al. 2020). Altered expression or mutations of neuroligin‐2 and its interacting partners have been associated with epilepsy, schizophrenia, autism, and anxiety (Ali et al. 2020). Furthermore, the activity‐dependent cleavage of neuroligin‐2 disrupts inhibitory synaptic function, leading to impaired inhibitory signaling and contributing to the hyperexcitability characteristic of epilepsy (Xu et al. 2024). These findings underscore the critical role of adhesion‐based mechanisms in regulating synaptic function and plasticity under both physiological and pathological conditions. Therefore, elucidating the molecular composition and dynamics of adhesion structures, especially SJs and PAJs, across the four types of synapses is of great importance.

Among PAJ components, nectin‐1 and nectin‐3 have been implicated in stress‐related cognitive disorders (Mandai et al. 2015; Mizutani et al. 2021). Nectin‐1 is upregulated in the synaptic fraction of the ventral hippocampus following contextual fear conditioning, where it contributes to fear memory consolidation, likely through its recruitment to PAJs (Fantin et al. 2013). Nectin‐3 expression in the CA3 is regulated by chronic stress via the corticotropin‐releasing hormone receptor 1, affecting spatial memory and dendritic complexity (Liu et al. 2019; X. D. Wang et al. 2013). Moreover, both nectin‐1 and nectin‐3 levels are altered in the prefrontal region (H. L. Wang et al. 2020), hippocampus (Liao et al. 2014; van der Kooij et al. 2014, 2015; X. D. Wang et al. 2013; Wei et al. 2015), and parahippocampus (Gong et al. 2018) following repeated exposure to severe stress during early postnatal or later life stages. These stress‐induced modulations suggest that nectin‐1 and nectin‐3 play important roles in stress‐induced neuroplasticity, particularly in memory formation and structural remodeling of hippocampal circuits.

In conclusion, our present study extends previous observations by revealing that PAJs are also present at *E*→*I*, *I*→*E*, and *I*→*I* synapses in vitro. In vivo, we demonstrate their presence at *E*→*E* synapses throughout the hippocampus and at a subset of *I*→*I* synapses in the CA1. This observation raises the possibility that PAJs are preferentially associated with specific *I*→*I* synapse subtypes, potentially reflecting functional heterogeneity, and more broadly supports the idea that PAJ components are differentially distributed across multiple synapse types in the hippocampus. Given the suggested involvement of PAJ components in stress‐induced remodeling, further investigation into their regulation and function across distinct synapse types may advance our understanding of how synaptic adhesion molecules regulate circuit remodeling in response to stress and experience and how dysregulation of these processes may lead to vulnerability to neuropsychiatric conditions.

## Data Availability

All of the datasets analyzed and all of the reagents used or generated during this study are available from the corresponding author on reasonable request.
